# Using Finite Element Modeling in Bone Mechanoadaptation

**DOI:** 10.1007/s11914-023-00776-9

**Published:** 2023-02-18

**Authors:** Quentin A. Meslier, Sandra J. Shefelbine

**Affiliations:** 1 Department of Bioengineering, Northeastern University, 334 Snell, 360 Huntington Ave, Boston, MA USA; 2 Department of Mechanical and Industrial Engineering, Northeastern University, 334 Snell, 360 Huntington Ave, Boston, MA USA

**Keywords:** Bone, Finite element model, Mechanoadpatation

## Abstract

**Purpose of the Review:**

Bone adapts structure and material properties in response to its mechanical environment, a process called mechanoadpatation. For the past 50 years, finite element modeling has been used to investigate the relationships between bone geometry, material properties, and mechanical loading conditions. This review examines how we use finite element modeling in the context of bone mechanoadpatation.

**Recent Findings:**

Finite element models estimate complex mechanical stimuli at the tissue and cellular levels, help explain experimental results, and inform the design of loading protocols and prosthetics.

**Summary:**

FE modeling is a powerful tool to study bone adaptation as it complements experimental approaches. Before using FE models, researchers should determine whether simulation results will provide complementary information to experimental or clinical observations and should establish the level of complexity required. As imaging technics and computational capacity continue increasing, we expect FE models to help in designing treatments of bone pathologies that take advantage of mechanoadaptation of bone.

## Introduction

Finite element (FE) modeling is a mathematical representation of a structure that incorporates geometry, material properties, and loading conditions to understand the mechanical environment. Because bone adapts geometry and material properties to its mechanical environment, FE models have been widely used to explore the effects of loading on bone growth and fracture healing, predict fracture risk, and explore bone mechanoadaptation to loading conditions. This review presents a brief historical summary of the use of FE modeling in the context of bone mechanoadaptation, bone adapting to mechanical loading. (We do not cover models of endochondral ossification during growth or fracture healing nor the use of FE to predict fracture risk.) We explore why we use FE models, how we validate our models, the required complexity of the models, current limitations, and potential benefits for future applications.

## Historical Context

In 1972, Brekelmans et al. [1] presented a 2D model of a femur with less than 1000 triangular elements and showed stress distribution under different loading conditions. Loading conditions were determined by taking into account hip anatomy and center of gravity [2]. These 2D models explored heterogeneous material properties and the influence of trabecular bone heterogeneity on the stress distribution in the femoral head [3]. Additionally, a 2D model of the patella was used to investigate the relationship between trabecular bone architecture and stress distribution [4].

The first 3D bone finite element models examined stress distributions during slow walking in human femurs [5, 6]. Computational results were compared to ex vivo measurements using an extensiometer [5, 7] and strain gauges [6]. Numerous studies during this time used FE models for stress analysis and implant design. Huiskes and Chao detailed the first decade of applications of FE models between 1972 and 1982 [8]. At this time (early 1980s), models were limited by computational power, thereby limiting the complexity of bone geometry and material properties (heterogeneous, nonlinear, anisotropic).

Between the late 1980s and early 1990s, improvements in computational power and advancement in imaging capabilities allowed more complex FE model geometry and investigation of mechanical stimulus predicting changes in bone density, material properties, and architecture due to loading history [9–12]. Carter et al. implemented a time-dependent approach to simulate bone remodeling and predict changes in bone density under various loading conditions applied on a 2D femoral head model [13, 14]. Expanding this work in 3D, Fyhrie et al. [11] compared cancellous bone morphology following different effective stresses (Von Mises, strain energy density, spherical stresses) and showed that Von Mises stress cannot accurately predict bone apparent density. The development of computed tomography (CT) led to image-based finite element modeling that accounts for three-dimensional architecture [15]. Image voxels were directly converted to brick elements to create the model mesh [15, 16]. The development of imaging techniques was critical to the first subject specific FE-model in which both geometry and material properties were estimated based on CT scan data [17]. In their review, Huiskes and Hollister detailed progress in structure optimization, bone remodeling, and fracture studies between 1983 and 1993 [10].

In addition to imaged-based geometric meshes, automatic meshing algorithms also improved, in the late 1990s,to allow the meshing of complex structures [18]. Tetrahedral meshes are easily constructed automatically from complex 3D geometry; hexahedral meshes require more manual manipulation of the mesh and computational power, but offers a more mathematically stable element compared to tetrahedral meshes [19]. More complex material properties were also integrated into the models, specifically heterogeneity (i.e. correlation between CT attenuation coefficient and elastic modulus) [20–22] and non-linearity (i.e. poroelasticity) [23–25]. Imaging with micro-computed tomography (microCT) provided a method for obtaining the micro-structure of the trabecular architecture [15, 16, 26, 27]. Micro-finite element models improved the quality of trabecular bone architecture modeling and allowed computational assessment of bone material properties [21, 28]. MicroCT-based 3D finite element models became commonly used in the context of animal models of bone adaptation [26, 29–31] to study bone mechanical environment induced by the experimental setup [26, 32–36]. Development of in vivo microCT allowed researchers to acquire multiple scans of the same bone over time and identify regions of bone formation and resorption between scans [31, 32, 37] and link regions of adaptation to the mechanical environment [31, 38].

FE models enable studies of the distribution of complex mechanical stimuli at different scales of the bone structure: from the tissue [23, 25, 33, 36, 39–42], to the cellular level [43–47]. Multi-scale FE models have been used to better understand the mechanical environment experienced at the cellular level based on the estimated stimulus in the whole bone [48, 49]. Specifically, modeling of an idealized lacuna canaliculi network (LCN) provided the first insights into the mechanical environment around osteocytes [47]. Image-based FE modeling enabled a more representative LCN geometry of a single or multiple lacunae, with or without osteocytes [44–46, 50]

**Fig. 1:**
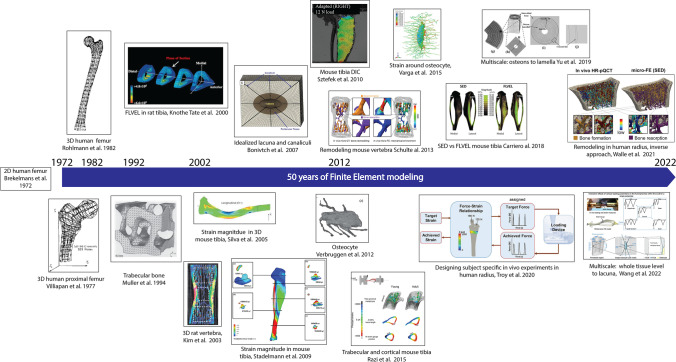
Evolution of FE models for bone adaptation within the last 50 years: from 2D human femur to 3D multi-scale models

To summarize, since 1972, increasing image resolution, computational resources, and advanced mechanical testing methods enabled more representative and complex bone FE models. FE models greatly contributed to the understanding of bone mechanoadpatation. Figure 1 illustrates the evolution of FE models used in bone adaptation studies over the past 50 years. The following section will detail recent uses of FE models in the context of bone adaptation.

## Why Do We Use FE Models in Bone Adaptation Studies?

FE models are used to estimate the bone mechanical environment under specific loading conditions. Simulation results can then be used to explain experimental measures of bone formation and resorption and to inform experimental design.

### To Explain Experimental Observations

In vivo animal models of adaptation explore how imposed mechanical loading or unloading results in bone formation and resorption. Typically, the applied mechanical loading is well-defined and controlled. FE models allow estimation of the mechanical environment within a region of interest [33, 42] that can be correlated to areas of bone adaptation [31, 38, 41]. Recently, mechanical environments have been compared between males and females [51], after ovariectomy [52, 53], after pharmaceutical treatment [54], and with genetic modifications [55, 56•].

Historically, stain magnitude was considered the mechanical stimulus triggering bone adaptation [57–59]. However, studies showed that other mechanical parameters such as strain rate, strain energy density, and fluid flow Could drive the adaptation [31, 59–63]. FE models have also been used to examine less commonly-considered stimuli such as the piezoelectric capacity of the bone [64•, 65]. FE models combined with remodeling algorithms can simulate changes in bone geometry and properties due to changes in their mechanical environment (i.e., application of external loads, insertion of implants). This approach involves hypotheses regarding the mechanical stimulus responsible for the adaptation (i.e., strain energy density, strain magnitude, fluid flow) and the adaptive response (i.e. change in structure or modulus). Typically remodeling algorithms use a mechanoadaptive theory [57, 66, 67]: when the mechanical signal of interest is above/below a certain threshold, bone formation/resorption is implemented according to the proposed algorithm [53, 68••, 69]. Studies have also implemented a lazy zone in which no formation or resorption occurs [63, 70•, 71, 72•]. In addition, response to heavy mechanical loading can be simulated to consider woven bone formation [73•] or damage [72•]. The adaptation that occurs in response to the stimuli is often modeled as a change in density or modulus [52, 70•, 74], or a change in bone geometry [68••, 73•]. Remodeling algorithms enable the prediction of bone adaptation under different loading conditions. Simulation results can then be compared to experimental results to assess the accuracy of the prediction [68••, 75••, 76••]. Because algorithms can be tuned to adjust the amount of bone formation (or increase in density or modulus), comparisons between in vivo experiments and computer models more often compare location of adaptive changes, which is much more dependent on the distribution of the stimulus. Thus, comparing computational and experimental results help to determine the mechanical stimuli that best predict adaptation. Using this approach, fluid flow was a better predictor of regions of adaptation than strain magnitude [63, 68••]. Using FE modeling and circuit theory, van Tol et al. [68••] estimated strain magnitude and load-induced fluid flow by taking into account the lacunar canalicular network and predicted the bone mechanoresponse. Computational bone formation and resorption predictions were compared to in vivo microCT measurements. Authors showed that fluid flow within the LCN predicted bone remodeling better than strain. Other models have used multi-scale stimuli. Goyal et al. used Strain Energy Density (SED) and diffusion of calcium ions as the stimuli driving adaptation under heavy mechanical loading to predict the formation of woven bone.

Table 1 details some studies in the past four years that have used remodeling algorithms in FE mechanoadpatation studies.

**Table 1:** Remodeling algorithm used in recent FE studies in the context of bone adaptation

Mechanical signal	Response simulated	References
Strain energy density	Change in bone density/modulus	Gonzalez et al., 2020 [70•] Anijs et al., 2022 [71] Park et al., 2022 [78] Poovarodom et al., 2022 [79]
	Change in bone density and geometry	Cheong et al., 2020 [52]
Strain gradient	Change in bone density and geometry	Du et al., 2021 [72•]
Stress/strain	Change in bone density/modulus	Levadnyi et al., 2021 [74]
	Tissue differentiation and changes in material properties	Mathai et al., 2022 [80]
Strain and ions flow	Woven or lamellar bone formation (geometry)	Goyal et al., 2022 [73•] Prasad et al., 2019 [69]
Fluid flow	Bone formation and resorption (geometry only)	van Tol et al., 2020
Electric charges (piezoelectric strain)	Change in bone density/modulus	Bansod et al., 2021 [64•]

Remodeling algorithms can be applied to cortical and trabecular bone. A recent review [81] detailed and compared computational models for trabecular bone remodeling.

In investigating bone remodeling, a typical approach is to apply a load on the model and simulate the bone formation and resorption. However, inverse approaches have also been developed to back-calculate the applied load based on the trabecular bone architecture and measured remodeling from animal experiments [69] and clinical data [82••].

Under the assumption of a specific driving mechanical stimulus, FE models can help identify regions of interest to further investigate the mechanism driving bone adaptation using dynamic histomorphometry, microCT analysis, and protein and gene expression analysis. Recently, Chlebek [83•] used FE simulation results to investigate gene regulation in bone regions experiencing different strain magnitudes and showed that, under loading, the transcriptomic response of cortical bone varied along the bone length, correlating with strain magnitude.

In all approaches discussed so far, a hypothesis regarding the stimulus driving adaptation is required. Thus, FE models provide only a potential explanation for in vivo measurement. One advantage of using models is the ability to explore multiple stimuli to see which match regions of bone formation most closely. For example, in a FE model of murine tibial loading model, strain energy density and fluid flow were examined and correlated to regions of bone formation [63, 77]. While strain energy density predicted formation on the periosteal surface, fluid flow predicted bone formation on both the endosteal and periosteal surfaces, as seen in the experiments. Because the mechanical stimuli are often related, contributions from different stimuli are challenging to tease apart. Indeed, increasing strain magnitude with the same loading frequency will result in an increased load rate, which also affects the fluid flow velocity. Thus, attributing bone formation only to changes in strain magnitude may provide an incomplete explanation of the experimental measurements [29, 35, 84].

### To Design and Inform

FE models are commonly used to inform the design of orthopedic implants. Appropriate bone adaptation around implants is critical to guarantee their integration. The mechanical behavior of prosthetics was one of the first applications of FE models in bone [10]. In their review, Taylor et al. [85] detailed the different models used for orthopedic applications until 2015. Today, FE models are still used in implant design. Levadnyi et al. used FE modeling, strain gages, and digital image correlation (DIC) and showed that the hip implant collar affects bone density over time [74]. Implant design and position can alter the bone adaptation around the prosthesis under loading. Thus, bone remodeling algorithms are also important to consider in the evaluation of prosthetics [74, 78, 79]. Investigations of dental implant positioning [79] showed that implant depth affects cancellous bone remodeling. FE models can also be used to predict bone ingrowth within the implant coating [80]. FE models of bone adaptation in prosthesis applications are often compared to clinical data [71].

In addition to implant design FE models can help in designing in vivo experiments, such that simulation results inform the loading protocol. Troy et al. used subject specific FE models of the human radius to inform their clinical experiments [86••]. They investigated the impact of strain magnitudes and strain rates on human bone adaptation, using a voluntary upper limb compressive task in healthy adult women. Subjects were assigned target forces based on FE simulation results to achieve low or high strain magnitudes and rates. This model was validated via multiple strain gauges measurement using ex vivo forelimb [87, 88]. They demonstrated that strain magnitude, rate, and number of applied loading bouts contribute to bone adaptation in healthy adult women.

In animal studies, FE models enable the design of loading conditions to help determine the loading parameters to apply in vivo. Eller et al. [89•] used a strain-matched approach to ensure equivalent strain magnitude in tibiae from control and high fat diet mice. High fat diet mice have significantly larger cortical bone cross sectional areas, requiring a larger load to obtain the same strain. Meslier et al. [90••] adapted this strain-matched approach to fluid flow. They used a mouse tibia model to estimate strain magnitude and fluid velocity in the cortical bone in response to various loading profiles. Simulation results were used to design their in vivo experiment, which aimed to dissociate the contribution of fluid flow compared to strain on bone adaptation.

FE models can be a powerful comparative tool to inform experimental design but is currently underutilized. We expect an increase in the use of FE models for protocol development in human and animal experiments in which the mechanical stimulus is challenging to measure in vivo non-invasively.

## How Complex Does the Model Need to Be?

With the development of imaging techniques and computational power, there is an increase in our ability to build more complex models. Model intricacy can be related to the geometry, materials properties, loading conditions, and the multi-scale aspect. However, the model complexity should be adapted to the research question.

### Geometry

One of the most common applications of FE modeling is to evaluate the distribution of mechanical stimuli in the model. The geometric complexity of the FE model influences the accuracy of the solution. Pickering et al. [91] compared beam theory and FE model of a mouse tibia to investigate load-induced strain distribution. They reported that by correcting the beam theory model with a loading correction factor, the estimation of strain distribution was comparable to the FE model. The correction factor must be adapted to the age, sex, and strain of the mice (i.e., calibrated). This work introduces the idea that simplified models that do not require advanced modeling skills can be used for simple estimation of the mechanical environment. However, beam theory can only be applied to the bone diaphysis and at the macroscopic level. Due to the curvature of most of the bones and specific features, having an accurate representation of the tissue geometry is important to obtain a representative distribution of the mechanical stimulus. For example, the fibula significantly impacts the strain distribution in the murine tibial bone [92], but adding a growth plate does not affect diaphyseal strains [93•].

Commonly, FE models are used to estimate the mechanical environment in the bone at a specific time point, therefore they do not take into account changes in geometry that could be induced by loading protocols [76••] or changes in material properties [94]. Using image-based models representative of different time points could address this limitation but such models are more labor intensive [76••, 95, 96•, 97]. When investigating the mechanical environment of the whole bone, considering only the cortical shell can help simplify the model. Nevertheless, depending on the research question, the trabecular bone can also be included [41, 98•].

In all FE models, the accuracy of the solution depends on the element size and a critical first step of modeling is to check for mesh convergence. Mesh density is of particular importance at material interfaces; gradients in material properties help to avoid stress concentrations.

### Material Properties

In most models, homogeneous material properties are assumed to simplify the model. However, bone is a heterogenous material, which can be taken into account in the model [68••, 76••, 99]. Heterogenous bone properties can lead to variation in the strain distribution [41, 55, 99]. To account for heterogeneity, Young moduli are based on attenuation coefficients from computed tomography images, which indicate bone density. Heterogeneous models can lead to small differences in strain distribution compared to homogeneous models, for example, in the mid-diaphysis of the mouse tibia [55]. However, in their micro FE model of the mouse tibia, Oliveiro et al. [100•] reported that homogenous models were the best compromise between accuracy and computational time to predict structural mechanical properties.

When FE models are used to study strain distribution in the bone, material properties are typically elastic. Characterization of fluid flow in the model requires consideration of the bone poroelastic properties. Viscoelastic properties are usually not taken into account but can be important in determining matrix deformation at different strain rates. Considering the viscoelastic properties of the pericellular matrix in the LCN could help distinguish the direct impact of fluid flow compared to the pericellular matrix deformation on osteocytes activation.

Current desktop computers have the computational power to model complex heterogeneous material properties and dynamic loading conditions. The limitation in modeling non-linear viscoporoelastic materials is in determining the bone viscoelastic and poroelastic properties from experiments.

### Loading Conditions

Loading conditions applied on the FE model often aim to emulate the load experienced within the experimental setup or during a typical physical activity such as walking. To do so, defined magnitudes and educated guesses on placement and direction are necessary. Loading conditions can be informed by imaging the sample during loading. Poulet et la [101] imaged the mouse tibia under uniaxial compression and showed the extreme flexed position made the femoral condyles contact with the posterior part of the tibia. Additional methods such as gait analysis, instrumented implants equipped with load sensors [102, 103], and musculoskeletal simulation software [104] help in defining physiological loading conditions [105, 106•]. Muscle forces can be included in the FE model as point loads or loads distributed over an attachment area. In some instances, muscle forces may not affect mechanical stimuli in the region of interest and can be neglected. For example, we found femoral muscles contribute significantly to the hip joint contact force, but do not affect stresses in the growth plate [126].

FE models have been recently used to estimate the influence of loading location in the mouse tibia on the strain distribution in the bone [93•]. Results suggested that in order to obtain a similar strain distribution, load location must be adjusted between specimens. Boundary conditions are assumed to be similar between computational models and in vivo setup and are expected to be reproducible between samples. To address the potential variability of load applied between samples, subject-specific FE models can be used for human studies [86••, 107] or in vivo imaging during loading for animal studies.

### Multi-scale

Osteocytes are mechanosensitive cells that play a crucial role in the bone adaptation. Characterizing the pericellular mechanical environment of osteocytes is critical for determining the mechanical stimuli necessary for activation. The mechanical environment around the osteocytes is challenging to assess experimentally and depends on the stimulus distribution and magnitude at the tissue level. Multi-scale models of bone adaptation have related the organ and tissue length scale to the cellular length scale during loading [48, 49, 108••, 109•, 110•]. FE models of bone lacunae demonstrated that the size and orientation of the lacuna affect the strain magnitude around osteocytes [111•]. A parametric study varying lacunar morphology (i.e., density, volume, equancy) and perilacunar bone properties indicated that increasing lacunar density resulted in increased deformation around the lacunae [112••]. Results from FE models that include lacunae suggest that alteration of lacunae morphology and properties due to aging or pathological processes might affect osteocyte mechanosensitive due to a change in their mechanical environment. Other parametric studies were conducted to examine the effect of permeability and porosity on the fluid flow [113] and showed that microstructural changes related to osteoporotic conditions could alter fluid flow around osteocytes. An osteocyte FE model has been used to investigate the effect of TGF-β deficiency and LCN degeneration on osteocytes’ mechanical environment [114••]. Results suggested that the expansion of the modeled pericellular space restored the mechanical environment around the aged osteocytes to young levels.

One of the challenging aspects of multi-scale modeling is linking the organ level to the lacuna canalicular system (LCS) and the osteocytes. These challenges were reviewed by Paul et al. [115]. In addition, FE modeling of strain and fluid flow at the cellular and LCS levels have been reviewed in detail [110•, 116••]. Recently, a multi-scale model was used to study the effect of multiple loading parameters on fluid flow in the lacuna canalicular network [108••]. Results from a continuum model were applied as boundary conditions to an idealized osteocyte lacuna. This multi-scale model demonstrated that strain magnitude and strain rate both affect the fluid velocity and shear stress around the osteocyte. However, these two stimuli do not seem to have an additive effect. Determining the mechanical environment at the cellular level will help to inform the remodeling algorithms.

## The Importance of Model Validation

Validation of finite element models is often performed by comparing simulation results to measured strain magnitudes using strain gages [55, 93•, 99, 108••, 117•, 118•, 119]. This method validates the estimated strain at single-point locations. Strain distribution is heterogenous in bones due to their complex shape and material properties. Thus, assumptions are necessary to extrapolate the validated area to the rest of the bone.

To address this limitation, digital image correlation (DIC) was used to validate FE models along the bone [120]. DIC allows researchers to characterize surface strain within a definite region of interest [74, 77, 120]. Expanding on this method, digital volume correlation (DVC) combined mechanical testing and microCT scan of undeformed and deformed bones allowing direct measurement of the strain in the tissue [100•, 121].

The precise strain magnitude values will vary between species, genotype, sex, and location on the bone due to differences in bone geometry, density, and material properties [122•]. Accurate estimation of a mechanical stimulus is critical for tuning experiments to the same stimulus across groups. However, an accurate measurement of the mechanical stimulus is not always possible nor necessary. For many experiments comparison across groups requires only higher/lower designation. For example, validation of simulated fluid flow velocity is complex. In Meslier et al., the model was validated for strain stimuli patterns with DIC but was used to compare high versus low fluid velocity in different loading conditions. An unvalidated model is likely sufficient for this purpose. FE models can estimate regions of high fluid flow velocity, explore the fluid flow dependence on material properties, allow a relative comparison between loading conditions, and identify the changes in the flow due to geometry alterations.

## Conclusion

FE is a powerful tool for investigating the distribution of complex mechanical signals in the bone under various experimental loading conditions and simulating the mechanoadaptive response with a change in material properties or geometry. FE models cannot supplant experiments. However, FE models can help to explain experimental results and to design experiments efficiently. Consideration of bone adaptation is critical to the design of implants and is facilitated by FE modeling. When embarking on modeling bone mechanoadaptation, it is essential to understand the research question clearly. Specifically, what will the model tell you that experiments cannot? Models should only be as complex as required by the research question; and unvalidated models may be sufficient for comparing across groups. As the model becomes more complex, more unknown variables are added, which may affect the model's ability to predict adaptive outcomes efficiently.

The FDA now considers finite element modeling a critical part of medical device design [123–125]. In the future, the FDA may accept simulation results as an important piece of evidence to indicate bone adaptation to implants or treatment. While the last 50 years of FE modeling have provided insight into mechanoadaptation of bone; the next 50 years will allow us to utilize this insight to design effective devices, therapies, and experiments to tap into the therapeutic potential of bone adaptation.
